# In Vitro Evaluation of Antimicrobial Activity of Minocycline Formulations for Topical Application in Periodontal Therapy

**DOI:** 10.3390/pharmaceutics12040352

**Published:** 2020-04-13

**Authors:** Jan-Luca Schmid, Martin Kirchberg, Sandra Sarembe, Andreas Kiesow, Anton Sculean, Karsten Mäder, Mirko Buchholz, Sigrun Eick

**Affiliations:** 1 Laboratory of Oral Microbiology, Department of Periodontology, School of Dental Medicine, University of Bern, CH-3010 Bern, Switzerland; jan.schmid@students.unibe.ch; 2 Institute of Pharmacy, Martin-Luther University Halle, D-06120 Halle (Saale), Germany; Martin.Kirchberg@pharmazie.uni-halle.de (M.K.); Karsten.Maeder@pharmazie.uni-halle.de (K.M.); 3 Characterization of Medical and Cosmetic Care Products, Fraunhofer Institute for Microstructures and Materials IMWS, D-06120 Halle/Saale, Germany; sandra.sarembe@imws.fraunhofer.de (S.S.); andreas.kiesow@imws.fraunhofer.de (A.K.); 4 Department of Periodontology, School of Dental Medicine, University of Bern, CH-3010 Bern, Switzerland; anton.sculean@zmk.unibe.ch; 5 Drug Design and Target Validation, Fraunhofer Institute for Cell Therapy and Immunology IZI-MWT and PerioTrap Pharmaceuticals GmbH, D-06120 Halle/Saale, Germany; mirko.buchholz@periotrap.com

**Keywords:** controlled release, periodontitis, local antibiotics, in vitro model, gingival flow

## Abstract

Periodontal therapy using antimicrobials that are topically applied requires slow or controlled release devices. The in vitro antimicrobial activity of biodegradable polymer formulations that contain a new minocycline lipid complex (P-MLC) was evaluated. The new P-MLC formulations that contained 11.5% minocycline were compared with pure minocycline or an existing commercial formulation, which included determination of minimal inhibitory concentration (MIC) values against two oral bacteria and activity on six-species periodontal biofilm. Moreover, the flow of gingival crevicular fluid (GCF) was modeled up to 42 days and the obtained eluates were tested both for MIC values and inhibiting biofilm formation. In general, MICs of the P-MLC formulations were slightly increased as compared with pure minocycline. Biofilm formation was clearly inhibited by all tested formulations containing minocycline with no clear difference between them. In 3.5 day old biofilms, all formulations with 250 µg/mL minocycline decreased bacterial counts by 3 log10 and metabolic activity with no difference to pure antimicrobials. Eluates of experimental formulations showed superiority in antimicrobial activity. Eluates of one experimental formulation (P_503_-MLC) still inhibited biofilm formation at 28 days, with a reduction by 1.87 log10 colony forming units (CFU) vs. the untreated control. The new experimental formulations can easily be instilled in periodontal pockets and represent alternatives in local antimicrobials, and thus warrant further testing.

## 1. Introduction

Periodontal disease is associated with an imbalance of microorganisms in subgingival biofilm. Bacteria highly present in these biofilms are *Porphyromonas* spp. and *Tannerella forsythia* [1]. *Porphyromonas gingivalis* has been designated as a keystone pathogen by modifying the biofilm towards a pathogenic one via modulation of the host response [2]. The response of the periodontium to inflammatory changes resulted in the loss of attachment and periodontal integrity [3].

Removal of the subgingival biofilm, either by manual or ultrasonic instrumentation, is an essential outcome of periodontal therapy and decreases the bacteria count associated with periodontal disease. When applied as a sole method, failures arise with increasing probing depths (≥5 mm) [4]. Systemic antibiotics improve the clinical outcomes, although their use should be restricted to severe cases [5], as antimicrobial use affects the gut microbiota [6] and is clearly associated with global development of antimicrobial resistance [7]. 

In particular, in supportive periodontal therapy, local antimicrobials are applied to treat residuals pockets [8]. Topical application can lead to a high concentration of the antimicrobial which is active in the diseased area and selectively targets microorganisms in the diseased area [9,10]. Systemic side effects can be reduced and application of controlled compliance by the periodontist to the patient is guaranteed [9,10]. 

However, an important problem is the high flow rates that are ≥20 µL/h of the gingival crevicular fluid (GCF) [11]. In order to avoid rapid depletion of the therapeutics and to keep them in place, sustained or controlled release devices are needed [9]. Desirable properties of the devices include easy application, biodegradability, and a continuous release of an antimicrobial-active substance over a defined time [9,10]. 

In so doing, topical antimicrobials combined with mechanical therapy can improve the clinical outcome [8]. This is underlined by a systemic review including more than 50 studies, in which the effect was most pronounced in deep and residual pockets [12]. 

Frequently, tetracyclines and analogs are used as local antibiotics [12]. Tetracylines (e.g., tetracycline, minocycline, and doxycycline) block bacterial growth by inhibiting protein synthesis [13]. Moreover, independent of the antimicrobial activity, they are frequently used to inhibit matrix metalloproteinases activities [14]. 

Several antibiotic formulations are on the market, such as gels containing doxycycline [15,16] or PLGA (poly-(lactide-*co*-glycolide)) based minocycline microspheres [17,18]. For the application of minocycline microspheres, the cartridge is connected with a special handle mechanism and used to insert the unit-dose cartridge into the base of the periodontal pocket, then, the thumb ring in the handle mechanism is pressed to expel the powder [19]. The minocycline microspheres have been proven in several clinical studies where the adjunctive used for mechanical debridement was compared with mechanical debridement only. A large clinical trial including more than 700 patients found minocycline microspheres highly efficient in probing depth reduction up to nine months [18]. Another study that included 127 subjects showed favorable results on the reduction of *P. gingivalis*, *T. forsythia*, and *Treponema denticola* [20]. Moreover, the adjunctive use of minocycline microspheres was able to improve the clinical outcome in the treatment of peri-implantitis [21]. 

**Figure 1:**
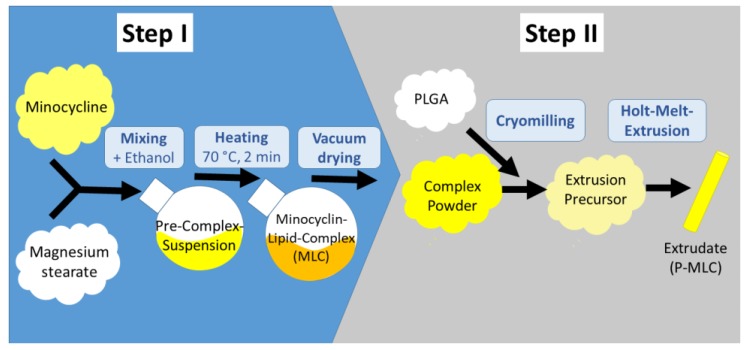
The investigated minocycline lipid complex (P-MLC) systems are manufactured in two sequential main steps. Step 1 includes the formation of the lipophilic minocycline lipid complex MLC by solvent and heat associated complex formation between minocycline and magnesium stearate. After evaporation of the intermediate solvent ethanol, the MLC complex is micronized, mixed with the biodegradable polymer poly-(lactide-*co*-glycolide) (PLGA), and extruded. As a result, a polymer-lipid complex extrudate P-MLC is obtained (diameter 600 µm). Two different PLGA polymers were used P_502_-MLC and P_503_-MLC.

In the present study, we investigated new experimental biodegradable formulations in the form of the extrudates, which could easily be placed into a periodontal pocket using a pair of tweezers. Recently, we described the manufacturing of new lipophilic complexes of tetracyclines, and their incorporation into these extrudates (Figure 1) [22].

The new formulations show higher stability in an aqueous environment as compared with the pure drug molecules [22]. This is an important aspect, as drug molecules in biodegradable PLGA polymers could be exposed to a very acidic aqueous microenvironment (pH 2) for longer times prior to their release [23]. This exposure could lead to drug inactivation by hydrolysis or the formation of covalent amide bonds [24]. The main focus of the first publication was on the complex formation, the analytical characterization, and the drug release kinetics from polymer-lipid complex extrudates. Although the principal bioactivity of the lipophilic complexes has been shown in preliminary experiments [22], much more detailed studies are necessary to explore the potential of the new formations with regard to its biological performance. Therefore, this study aimed to investigate the time dependence of the antimicrobial activity in relevant in vitro models. In particular, the dilution by the GCF flow was mirrored. The newly developed polymer-lipid complex extrudates were compared with a commercially available product and minocycline as the drug substance.

## 2. Materials and Methods 

### 2.1. Antimicrobials

The manufacturing and characterization of the PLGA and minocycline lipid complex (P-MLC) extrudates have been described in a previous publication [22]. In short, for both used formulations (P_502_-MLC and P_503_-MLC), minocycline (Ontario Chemicals Inc., Guelph, ON, Canada) was chelated with calcium and preferably magnesium stearate (both Magnesia Germany, Müllheim, Germany) in the molar ratio 1:2. Subsequently, the complex was mixed with the desired PLGA polymer (Evonik, Darmstadt, Germany) and cryomilled. This composition was utilized for the hot melt extrusion with a 600 µm device. Each of the extrudates contained 11.5% (m/m) of minocycline [22]. 

Positive controls were minocycline as a substance (Ontario Chemicals Inc., Ontario, ON, Canada) and minocycline microspheres (Arestin^®^; OraPharma, Bridgewater, NJ, USA). As a negative control, the drug free PLGA/lipid extrudate (P_503_ and of magnesium stearate; vehicle) was included. All minocycline formulations were also kept thoroughly in the dark throughout the experiments. 

To achieve a homogeneous distribution of the formulations in the assays, the extrudates and the microspheres were kept at −20 °C overnight in a freezer, and thereafter micronized with a pestle in a porcelain mortar. 

### 2.2. Microorganisms

In the assays, the following six bacterial strains were used: *Porphyromonas gingivalis* ATCC 33277, *Tannerella forsythia* ATCC 43037, *Fusobacterium nucleatum* ATCC 25586, *Streptococcus gordonii* ATCC 10558, *Actinomyces naeslundii* ATCC 12104, and *Parvimonas micra* ATCC 33270. All strains were precultured on tryptic soy agar plates (Oxoid, Basingstoke, GB) with 5% sheep blood for 24 h at 37 °C with the respective conditions (*S. gordonii*, *A. naeslundii* with 10% CO_2_, others anaerobically). 

The determinations of minimal inhibitory concentration (MIC) values were made only against *P. gingivalis* ATCC 33277 and *S. gordonii* ATCC 10558. 

All six strains were included in the biofilm assays. For that, bacteria were suspended in phosphate buffered saline (PBS) according McFarland 0.5 (about 1.5  × 10^8^ microorganisms) and mixed in a ratio 1:2:4 (*S. gordonii*)/(*A. naeslundii*)/(each other strain). 

### 2.3. Determination of Minimal Inhibitory Concentration (MIC)

A two-fold dilution series of the formulations dissolved in Aqua Dest was prepared. The highest concentration of the formulations was always equivalent to 64 µg/mL minocycline. The vehicle was adapted to the amount of this concentration of the test formulation. Thereafter, each 100 µL of test substance solution followed by 100 µL of bacterial suspension (about 10^6^ bacteria/mL) in doubled concentrated Wilkins–Chalgren broth (Oxoid) with 10% of lysed horse blood and 10 µg/mL β-NAD (Merck KGaA, Darmstadt, Germany) was pipetted per well of a 96-well-plate. The final tested concentrations of the formulations ranged from 32 µg/mL to 0.125 µg/mL minocycline. After an incubation time of 18 h (anaerobes 42 h) in respective atmosphere, the MIC was assessed as the lowest concentration without visible growth (turbidity). 

### 2.4. Activity on Biofilm Formation

First, the test formulations were dissolved in Aqua Dest equivalent to 125 µg/mL. Then, the wells of a 96-well-plate were coated with 10 µL/well for 1 h, before 10 µL/well PBS with 1.5% bovine serum albumin (PBS/SA) was added for 10 min followed by the mixed bacterial suspension in Wilkins–Chalgren broth with 5% of lysed horse blood and 5 µg/mL β-NAD. Here, the total counts of colony forming units (CFU) of the biofilms were counted after 6 h and 24 h incubation in an anaerobic atmosphere at 37 °C. After 24 h of incubation, the quantity and metabolic activity of the biofilms were also measured. The quantification was made by staining with crystal violet according to recently published protocols [25,26]. In short, after washing, the biofilms were fixed at 60 °C for 1 h. Thereafter, 50 µL of 0.06% crystal violet solution was given per well. After 10 min of incubation at room temperature, the stained biofilms were dissolved by 200 µL of 30% acetic acid, and after transferring to a new microtiter plate, the OD_600nm_ was measured. Biofilm metabolic activity was assessed using resazurin as a redox indicator [25,27]. After short washing of the biofilms, 100 µL of nutrient broth containing 0.06 mM resazurin (Merck KGaA) per well was added. After 1 h of incubation at 37 °C, the plate was measured at 570 nm against 600 nm. 

### 2.5. Activity on an Already Formed Biofilm

Biofilms using the multispecies mixture consisting of 6 species were formed for four days. Nutrient broth was exchanged and selected bacteria (*P. gingivalis*, *T. forsythia*) were added again after 2.5 days. At four days of biofilm formation, after removing the nutrient media and short washing with PBS, 100 µL of test substances solutions in a concentration equivalent to 1000, 500, and 250 µg/mL were added per well for 10 min, before 100 µL of nutrient broth were pipetted per well (final concentrations of minocycline 50, 250, and 125 µg/mL). After an overnight incubation (18 h), the CFU, biofilm quantity, and biofilm metabolic activity were determined, as described before. 

The Biofilm experiments were made in two independent experiments in each independent quadruplicate. ANOVA with post hoc Bonferroni was used for statistical analysis. The level of significance was set to *p* = 0.05. Software SPSS 25.0 (IBM SPSS Statistics, Chicago, IL, USA) was used.

### 2.6. Simulation of the Release Kinetic in a Periodontal Pocket

The GCF flow was simulated in a model. The basis of the experimental setup was a resting volume of 1.5 µL with a flow of 44 µL/h in periodontitis, which decreased to 15 µL/h 12 weeks after therapy (Goodson et al., 2003). Thus, formulations equivalent to 1 mg minocycline were put into tubes with 23.5 μL PBS/SA. After 30 min, tubes were centrifuged at 5000× *g* for 1 min and 22 µL were removed and replaced by fresh 22 µL PBS/SA. Then, at diverse time points, the exchange of the eluates using different volumes was repeated (Table 1). All obtained eluates were stored at −20 °C until analysis. 

For MIC determination, eluates were used which were obtained at 1, 2, 4, 6, 24 h, and 2, 4, and 7 days, and then weekly up to 42 days. A two-fold dilution series of the eluates was prepared. Eluates were handled as the dissolved formulations before in the assays. The highest dilution of the eluate inhibiting growth of *S. gordonii* and *P. gingivalis* was recorded. 

Furthermore, to assess the potential activity on the biofilm formation, eluates obtained at 24 h, and 2, 7, 14, and 28 days were used. The wells of a 96-well plates were coated with 10 µL eluate/well for 1 h. The further procedure of biofilm formation was as mentioned before. However, here, the CFU were counted after only 6 h of incubation. 

## 3. Results

### 3.1. Antimicrobial Activity of the Formulations Against Planktonic Bacteria

The minimal inhibitory activity of the P-MLC formulations was slightly less, but in the range of the pure substance. The difference was one to two dilution steps related to the pure substance. The microspheres were equally active as the substance. The minocycline free vehicle did not exert any antibacterial activity against the tested bacteria (Table 2). 

### 3.2. Activity of the Formulations on Biofilms

**Figure 2:**
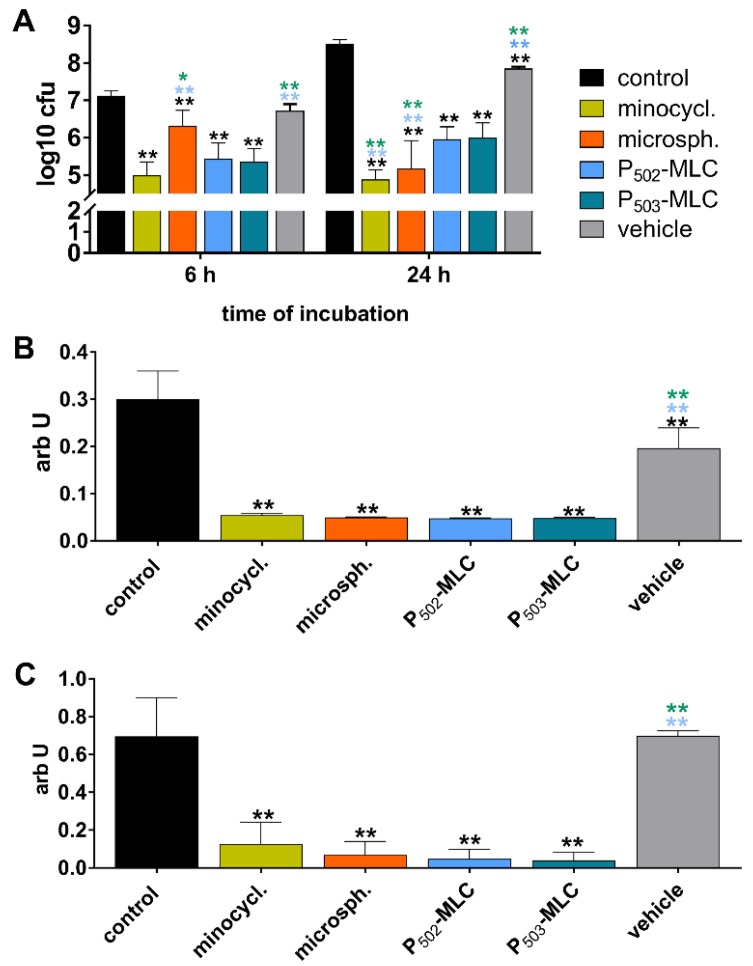
Activity of the minocycline formulations (substance (minocycl.) and microspheres (microsph.) polymer-lipid complex extrudate (PLM-C)) and PLM-C without minocycline (vehicle) on formation of a six-species biofilm. Bacterial counts determined as colony forming units (CFU) (**A**); biofilm quantity (**B**); and metabolic activity (**C**). ** (black) *p* < 0.01 vs. control; ** (blue) *p* < 0.01 vs. P_502_-MLC; * (green) *p* < 0.05 / ** (green) *p* < 0.01 vs. P_503_-MLC.

All tested experimental formulations and positive controls clearly inhibited biofilm formation (Figure 2). Regarding the CFU counts, the pure substance was the most active, with a reduction of 2.1 log10 after 6 h and 3.6 log10 after 24 h. The biofilm quantity was about one-sixth and the metabolic activity was about one-sixth, one-tenth related to the controls, here, no difference among the formulations with minocycline occurred, but the vehicle also reduced the CFU counts by 0.6 log10 and the biofilm quantity by about one-third. 

### 3.3. Activity on an Already Formed Biofilm

**Figure 3:**
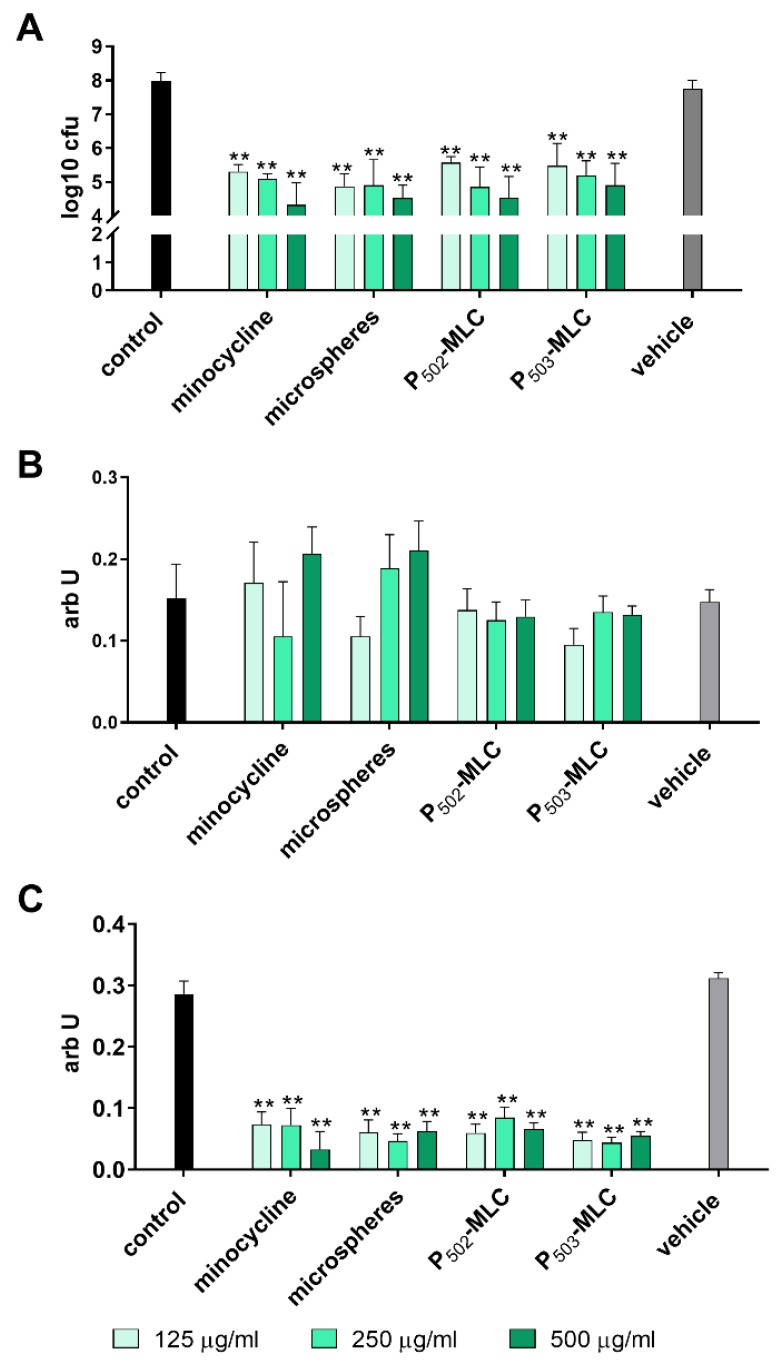
Activity of the minocycline formulations (substance (minocycl.) and microspheres (microsph.) polymer-lipid complex extrudate (PLM-C)) and PLM-C without minocycline (vehicle) on a six-species biofilm formed over 3.5 d. Bacterial counts determined as colony forming units (CFU) (**A**); biofilm quantity (**B**); and metabolic activity (**C**). ** *p* < 0.01 vs. control.

Analyzing the activity on an already formed biofilm, the reduction of the CFU counts was between 2.4 and 3.7 log10 CFU with a tendency of more activity with the higher concentrated minocycline formulations. Among the minocycline formulations, there was no difference and metabolic activity was also clearly reduced by all minocycline formulations. No clear effect on biofilm quantity was visible. The vehicle did not show any activity on the established biofilm (Figure 3). 

### 3.4. Antimicrobial Activity of the Eluates on Planktonic Bacteria 

**Figure 4:**
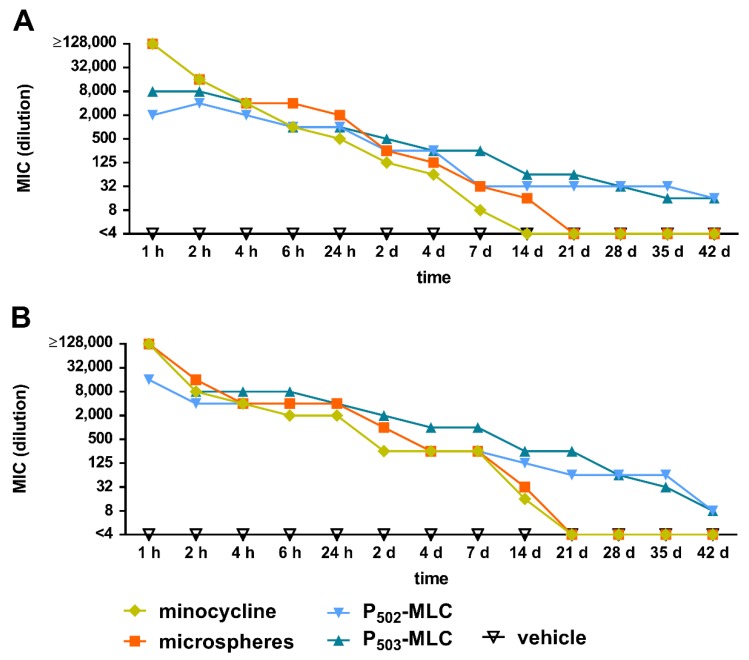
Minimal inhibitory concentration (maximum dilution) of the eluates obtained over a period of 42 days from minocycline formulations (substance (minocycl.) and microspheres (microsph.) polymer-lipid complex extrudate (PLM-C)) and PLM-C without minocycline (vehicle) according to 1 µg of minocycline and simulating the flow of the gingival fluid against *Streptococcus gordonii* ATCC 10558 (**A**) and *Porphyromonas gingivalis* ATCC 33277 (**B**).

Initially, eluates of all minocycline formulations inhibited the growth of *S. gordonii* ATCC 10558 and *P. gingivalis* ATCC 33277. As expected, the activity decreased continuously over time. At 28 days, there was no remaining activity for the pure drug substance and the microspheres, whereas the eluates of the P-MLC were still active after 42 days. The eluates of the vehicle did not have any activity at any time (Figure 4).

Considering the MIC value of the minocycline substance, the antimicrobial active concentration of minocycline was calculated. At 1 h, it was >16 mg/mL for the substance and the microspheres and 2 to 8 mg/mL for the P-MLC. At 24 h, the values decreased to 62.5 µg/mL for the substance, each 125 µg/mL for the P_502_-MLC formulation and the microspheres, and 250 µg/mL for the P_503_-MLC formulation. From 21 days, this concentration was less than 1 µg/mL for the substance and the microspheres, whereas the eluates of both P-MLC formulations had an activity of 4 µg/mL minocycline (Table S1). 

### 3.5. Antibiofilm Activity of the Eluates

**Figure 5:**
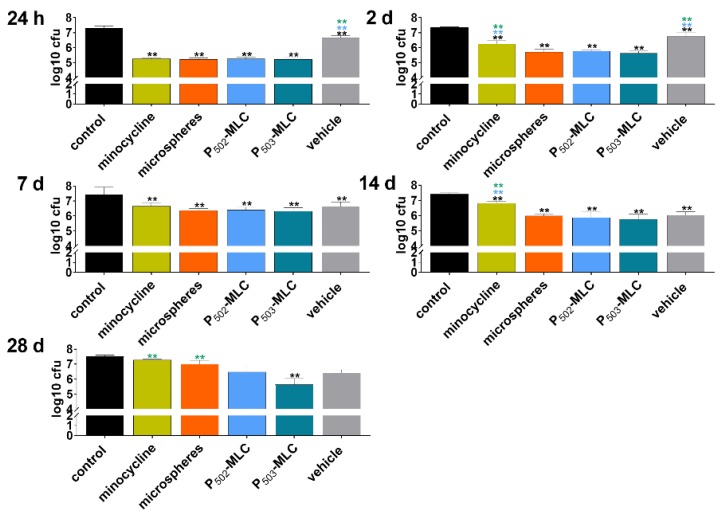
Activity of the eluates obtained over a period of 42 days from minocycline formulations (substance (minocycl.) and microspheres (microsph.) polymer-lipid complex extrudate (PLM-C)) and PLM-C without minocycline (vehicle) according to 1 µg of minocycline and simulating the flow of the gingival fluid on biofilm formation (CFU after 6 h). ** (black) *p* < 0.01vs. control; ** (blue) *p* < 0.01 vs. P_502_-MLC; ** (green) *p* < 0.01 vs. P_503_-MLC.

All eluates obtained from minocycline preparations at 1 day inhibited biofilm formation by about 2 log10 CFU. Later, up to 14 days, eluates only obtained from minocycline P-MLC or microspheres formulations reduced the CFU in biofilms by more than 1 log10. At 28 days, only the eluate obtained from P_503_-MLC showed a reducing effect (1.9 log10) and in addition, the eluates obtained from the vehicle decreased the CFU in biofilm by 1.4 log10 and 1.1 log10 at day 14 and day 21, respectively (Figure 5).

## 4. Discussion and Conclusions

In this study, newly developed formulations with magnesium stearate and PLGA as the vehicle were evaluated regarding their antimicrobial activity. Except for analyzing the formulations directly, the gingival flow was simulated, and eluates of the formulations obtained for a period of 42 days were investigated. A controlled long-lasting activity of the minocycline as the loaded antibiotic was confirmed.

The formulations were prepared as extrudates with a 600 µm device, of which the P_503_-MLC extrudate had a larger diameter than the P_502_-MLC extrudate due to viscoelastic properties [22]. For the experiments, they were available as cylinders and easy to cut to the necessary size. However, for clinical use, other sizes and shapes could also be created. 

Minocycline was selected because of the fact that it is one of the best documented local antibiotics in periodontal therapy [12]. In general, minocycline is also discussed as an anti-inflammatory drug for the treatment of Alzheimer’s disease [28] and a cardiovascular therapeutic agent related to its anti-inflammatory, antiapoptotic, and antioxidant properties [29]. 

As a local drug in periodontal therapy, different approaches have been taken, such as loaded in PLGA hydrogels [30], in in situ reversed hexagonal mesophase [31], or in liquid crystals [32]. In this study, the experimental formulations were compared with minocycline microspheres which have been commercially available for many years. 

First, experimental formulations were directly compared with pure minocycline and minocycline microspheres. A comparison of the different formulations showed that the pure substance was the most active against the two tested species. The experimental formulations exerted slightly less activity. The purpose of a controlled release device is that the antimicrobial activity is prolonged over time. Although the experimental formulations were dispersed as much as possible, it is suggested that minocycline was still covered by the vehicle substances. The total release of minocycline from these formulations was found to be only half of the minocycline magnesium and calcium stearate complexes [22]. 

Biofilm formation findings confirm the MIC results regarding the viable bacterial counts within the biofilm. Applying the formulations on an already formed biofilm, there was no visible difference between the formulations. Viable bacterial counts decreased by about 3 log10, but a biofilm eradication did not occur, although using high concentrations of the antibiotic were used over time. Once more, this finding underlines that antibiotics alone are not able to eliminate bacteria within the biofilm [33], and therefore, in periodontal therapy, their use is limited as adjuncts to instrumentation [34]. 

To mimic release kinetics in in vitro assays, in most cases the controlled release device is placed in defined, constant volumes over time [35,36,37]. In order to simulate the clinical situation as closely as possible, a GCF flow during a periodontal therapy with decreasing volumes [11] was simulated. Thus, the initial volume was adjusted to those of the periodontal pocket and the exchange was simulated accordingly taking into account the reduced GCF flow during periodontal therapy. Regarding its protein composition, since the GCF closely resembles a diluted serum [38], a buffered saline solution supplemented with serum albumin was added. The obtained eluates were used for analysis of the antimicrobial activity, up to 42 days. However, limitations of the in vitro assay have to be addressed. A short centrifugation step was included mainly for the purpose of sedimenting condensed water in the tubes. In particular, at the beginning of the experiments (when having only small volumes), an effect on the release of the minocycline into the environment cannot be completely excluded. In addition, the method did not consider a potential attachment and uptake of the antibiotics by epithelial cells, fibroblasts, and immune cells. Minocycline uptake by oral epithelial cells has been reported by [39]. Further accumulation in gingival fibroblasts has been shown [40]. Tetracycline analogs seem to be adsorbed mainly by plasma proteins and, when administered orally, their concentrations in GCF were found to be low, in about 50% they did not reach 1 µg/mL [41]. However, the concentration of tetracycline can reach more than 1000 µg/mL in the periodontal pocket when applied as a non-resorbable fiber over a few days [42]. For doxycycline, another tetracycline analogous, the concentrations in the periodontal pocket were about 1000 µg/mL after 2 h of application of the formulations containing either 8.5% or 14% of doxycycline, and then the concentrations decreased to 8 and 19 µg/mL after 12 days [43]. 

In the present study, the in vitro antimicrobial activity of the newly developed formulations lasted up to 42 days, while the activity of the minocycline microspheres was detectable only up to 21 days. Unfortunately, concerning the concentration or antimicrobial activity of minocycline, after applying minocycline microspheres in the periodontal pockets, exact data are not available. It is only mentioned once that the concentration was about 340 µg/mL in the GCF 14 days after application [18]. Differences between the eluates of the two experimental formulations were seen regarding their direct inhibitory activity on bacterial growth, as well as on retarding biofilm formation. The P_503_-MLC was more active than the P_502_-MLC. The higher activity could be related to the larger diameter [22] and the resulting higher surface which allows an initial faster release of the antibiotic. 

The biofilm experiments showed that the vehicle itself (both directly and the obtained eluate) was able to inhibit the biofilm formation. The vehicle contained PLGA and magnesium stearate. PLGA is often incorporated in local antibiotics formulations [44,45]. However, a direct antimicrobial activity of the vehicle was not found which is in accordance with studies using PLGA nanoparticles [45,46]. It has also been reported that unloaded PLGA-nanoparticles did not affect biofilm formation of *Pseudomonas aeruginosa* [46,47]. It is suggested that the inhibition of biofilm formation is linked to the incorporated magnesium stearate. In particular, magnesium is being discussed as an implant material, it exerts antibacterial activity against planktonic *Staphylococcus epidermidis*, *Staphylococcus aureus*, and *Escherichia coli*, furthermore, it prevents biofilm formation of these species [48]. In addition, magnesium oxide nanoparticles clearly inhibited the growth of Staphylococci, *E. coli*, *Pseudomonas aeruginosa,* and *Candida* ssp. in a study by [49] which also demonstrated a disruption of a nascent biofilm by magnesium oxide nanoparticles [49].

In conclusion, the tested controlled release device loaded with minocycline (especially P_503_-MLC) seems to be a suitable alternative to commonly used systems, thus warranting further testing in animal models, and thereafter in clinical trials.

## 5. Patents

The hereby presented work was filed in a patent application, which was registered under PCT/EP2019/079566 at the EPA dated on 2019/10/29 as a community invention of Fraunhofer-Society, Martin-Luther-University Halle-Wittenberg and the University of Bern.

## Figures and Tables

**Table 1:** Scheme of pipetting to simulate the gingival flow.

Time	PBS/SA Per Tube with Antimicrobial (1 mg)	
	Removal (µL)	Addition (µL)
T 0	-	23.5
T 30 min	22	22
T 60 min	22	44
T 2–4 h each h	44	44
T 4 h	44	88
T 6 h	44	792
T 24 h	792	1056
T 2 d–T 3 day each day	1056	1056
T 4 day	1056	3168
T 7 days	3168	3500
T 10.5 day	3500	3500
T 14 days	3500	3304
T 17.5 day	3304	3304
T 21 days	3304	3108
T 24.5 day	3108	3108
T 28 days	3108	2912
T 31.5 day	2912	2912
T 35 day	2912	2716
T 39.5 day	2716	2716
T 42 days	2716	-

**Table 2:** Minimal inhibitory concentrations of the minocycline formulations (equivalent to µg/mL minocycline).

Formulation	*S. gordonii* ATCC 10558	*P. gingivalis* ATCC 33277
Minocycline	0.5	0.25
Microspheres	0.5	0.25
P_502_-MLC	1	0.25
P_503_-MLC	2	0.5
Vehicle	No inhibition	No inhibition

## Supplementary Materials

The following are available online at https://www.mdpi.com/1999-4923/12/4/352/s1, Table S1: Antimicrobially active concentration of minocycline (µg/mL) in eluates obtained from minocycline P-MLC or microspheres formulations over a period of 42 days.
