# Supplementary material for: In Vitro Evaluation of Antimicrobial Activity of Minocycline Formulations for Topical Application in Periodontal Therapy
Source: Pharmaceutics. 2020 Apr 13;12(4):352. doi: 10.3390/pharmaceutics12040352 (PMC7238147; doi:10.3390/pharmaceutics12040352)
Supplement: Supplementary file 1 [file pharmaceutics-12-00352-s001.pdf]

pharmaceutics

MDPI

Supplementary Materials: In Vitro Evaluation of Antimicrobial Activity of Minocycline Formulations for Topical Application in Periodontal Therapy

Jan-Luca Schmid, Martin Kirchberg, Sandra Sarembe, Andreas Kiesow, Anton Sculean, Karsten Mäder, Mirko Buchholz and Sigrun Eick

Table S1. Antimicrobially active concentration of minocyclin (µg/mL) [1] in eluates obtained from minocycline P-MLC or microspheres formulations over a period of 42 d.

	Substance	Microspheres	P_{502}-MLC	P_{503}-MLC
**1 h**	>16000	>16000	2000	8000
**2 h**	4000	4000	1000	2000
**4 h**	1000	1000	1000	2000
**24 h**	250	1000	500	1000
**2 d**	62.5	125	125	250
**7 d**	31.3	31.3	31.3	125
**14 d**	<2	8	15.6	31.3
**21 d**	<1	<1	15.6	31.3
**28 d**	<1	<1	15.6	15.6
**35 d**	<1	<1	15.6	8
**42 d**	<1	<1	4	4

[1] Calculation was based on the active maximum dilution of eluates against *S. gordonii* ATCC 10558 and *P. gingivalis* ATCC 33277 and their respective MIC values.
